# Sublingual bivalent vaccination with PspA-C-CPE and Alcaligenes lipid A induces protective immunity against both Streptococcus pneumoniae and Clostridium perfringens

**DOI:** 10.21203/rs.3.rs-10001561/v1

**Published:** 2026-07-01

**Authors:** Yuki Hirayama, Ken Yoshii, Atsushi Shimoyama, Keigo Iemitsu, Eri Node, Hiroshi Kiyono, Masuo Kondoh, Koichi Fukase, Jun Kunisawa

**Affiliations:** National Institutes of Biomedical Innovation, Health, and Nutrition (NIBN); National Institutes of Biomedical Innovation, Health, and Nutrition (NIBN); The University of Osaka; National Institutes of Biomedical Innovation, Health, and Nutrition (NIBN); National Institutes of Biomedical Innovation, Health, and Nutrition (NIBN); Chiba University; The University of Osaka; The University of Osaka; National Institutes of Biomedical Innovation, Health, and Nutrition (NIBN)

## Abstract

*Streptococcus pneumoniae* and *Clostridium perfringens* are major pathogens causing severe respiratory infections and foodborne illnesses, posing critical threats particularly in communal settings. Current countermeasures face significant limitations, including restricted serotype coverage for *S. pneumoniae* and the lack of approved vaccines for *C. perfringens* enterotoxin (CPE). Effective vaccines tailored to these pathogens are urgently needed. Promising candidates include pneumococcal surface protein A (PspA) for broad serotype coverage and the C-terminal receptor binding domain of CPE (C-CPE). The novel sublingual vaccine strategy we present here combines a recombinant bivalent fusion protein PspA-C-CPE with *Alcaligenes*lipid A, a potent mucosal adjuvant. Sublingual administration of this combination vaccine elicited robust and long-lasting protective immunity in mice. This vaccine effectively neutralized circulating CPE to prevent lethal hyperkalemia and promoted the rapid clearance of *S. pneumoniae* from the lungs after lethal challenge. Furthermore, *Alcaligenes* lipid A enhanced the production of IgG against both antigens for at least 49 weeks. These findings demonstrate that sublingual administration of PspA-C-CPE and *Alcaligenes*lipid A can orchestrate simultaneous defense in the mucosal and systemic compartments. This non-invasive vaccine offers immense public health potential for providing enduring comprehensive, concurrent protection against pneumococcal respiratory infections and CPE-associated foodborne illnesses.

## Introduction

Mucosal tissues like the respiratory and gastrointestinal tracts serve as critical barriers against the invasion of exogenous pathogens. For example, *Streptococcus pneumoniae* is a leading cause of pneumonia, meningitis, and otitis media and leads to high mortality rates even in modern society [1]. In contrast, *Clostridium perfringens* is well known to cause foodborne illness characterized by diarrhea, which is mediated by *C. perfringens* enterotoxin (CPE) [2]. These two pathogens are highly prone to transmission and dissemination within communal living environments, such as hospitals, schools, and aged care facilities. Furthermore, they present significant clinical challenges due to the high risk of severe disease progression, particularly in young children and elderly adults with underlying comorbidities [3, 4]. Therefore, vaccines that protect against these pathogens would provide important public health benefits.

However, current countermeasures against these pathogens face significant limitations. Available polysaccharide and conjugate vaccines against *S. pneumoniae* are restricted to specific serotypes, covering at best only 23 of over 100 identified strains. In addition, current *S. pneumoniae* vaccines require complex, high-cost manufacturing processes [5–7]. To achieve broader coverage, pneumococcal surface protein A (PspA), a highly conserved antigen expressed on virtually all isolates, has emerged as a promising candidate for a universal pneumococcal vaccine [8, 9]. Regarding *C. perfringens*, despite its substantial public health impact, no approved vaccine currently exists. *C. perfringens* pathogenesis is driven by the non-toxic C-terminal receptor binding domain of CPE (C-CPE), which binds to claudin-4 on the intestinal epithelium, enabling the N-terminal CPE domain to form pores that disrupt the epithelial barrier [2, 10]. Thus, inducing neutralizing antibodies against C-CPE is a rational preventive strategy. However, C-CPE itself exhibits poor immunogenicity, hindering the development of an effective standalone subunit vaccine [11, 12].

To overcome these clinical and manufacturing challenges, we focused on multivalent vaccines capable of conferring protection against distinct infectious diseases through a single vaccine formulation. Among such strategies, recombinant fusion proteins offer a particularly attractive approach by integrating multiple antigens into a single molecular entity. We previously demonstrated that fusing the poorly immunogenic C-CPE with a highly immunogenic antigen yields an effective bivalent vaccine, successfully inducing protective immunity against both corresponding pathogens [11–13]. In addition, the manufacture of conventional multivalent vaccines requires the individual purification and subsequent admixture of component antigens, thereby driving high costs due to complex production processes and stringent quality control requirements [14]. In contrast, an approach using a single recombinant fusion protein represents a highly practical strategy that substantially simplifies both manufacturing and quality control, thereby reducing overall production costs. Building on this rationale, we hypothesized that a recombinant fusion protein combining PspA and C-CPE (PspA-C-CPE) could serve as a highly effective bivalent antigen.

Although most currently licensed vaccines are administered parenterally, they primarily induce systemic IgG responses and generally fail to elicit secretory IgA, which is essential for mucosal defense. Therefore, mucosal vaccination has emerged as an effective strategy to confer dual protection in both systemic and mucosal compartments. Sublingual administration has garnered particular attention recently [15]. Antigens administered sublingually rapidly penetrate the non-keratinized stratified squamous epithelium and activate immune cells in the lamina propria. These activated cells subsequently migrate via the common mucosal immune system, enabling sublingual vaccination to elicit broad and robust immune responses at distant mucosal sites [16, 17]. However, even with the strategic advantages of multivalent fusion proteins, the sublingual administration of recombinant subunit antigens typically requires a potent mucosal adjuvant to induce effective immune responses [18].

Our previous studies revealed that lipopolysaccharide derived from *Alcaligenes faecalis*, a commensal bacterium residing in gut-associated lymphoid tissues such as Peyer’s patches, acts as a weak Toll-like receptor 4 agonist [19, 20]. We also demonstrated that its immunologically active core, lipid A, exerts optimal immunostimulatory activity, positioning it as an effective and safe mucosal adjuvant candidate [21]. Indeed, the sublingual administration of *Alcaligenes* lipid A induced antigen-specific IgA and Th17 responses, conferring protective immunity against mucosal threats, such as *S. pneumoniae* infection and cholera toxin, while minimizing inflammation at the administration site [22].

In this study, we designed the recombinant bivalent fusion protein PspA-C-CPE. We then investigated whether sublingual immunization of PspA-C-CPE, combined with *Alcaligenes* lipid A as a mucosal adjuvant, successfully and simultaneously induced protective immunity against both pneumococcal infection and CPE-induced pathogenesis.

## Materials and Methods

### Mice

Female BALB/c mice (7 weeks old) were purchased from CLEA Japan (Tokyo, Japan). The mice were acclimated to the animal facility of the National Institute of Biomedical Innovation, Health, and Nutrition (NIBN; Osaka, Japan). All procedures in mice were performed under isoflurane (Forane; AbbVie, North Chicago, IL, USA) inhalant anesthesia. At the end of the experiments, the mice were killed by cervical dislocation under deep anesthesia. All animal experiments were conducted in accordance with the guidelines of the NIBN Animal Care and Use Committee and approved by the NIBN Animal Experiment Ethics Committee (approval numbers: DSR04–37R7, DSR04–38R7). The animal experiments in this study comply with the ARRIVE guidelines.

### Preparation of Alcaligenes lipid A

*Alcaligenes* lipid A was chemically synthesized according to a previous report [21] or purchased (Peptide Institute Inc., Osaka, Japan), dissolved in dimethyl sulfoxide (Nacalai Tesque, Kyoto, Japan), and stored at − 30°C.

### Preparation of recombinant protein and removal of endotoxin

The genes encoding PspA and C-CPE, and the fusion protein of the two (PspA-C-CPE), were amplified by polymerase chain reaction. Following previously reported methods [23, 24], we cloned these amplified products into the pET16b expression vector (Novagen, Darmstadt, Germany) to construct the expression plasmids pET16b-PspA, pET16b-C-CPE, and pET16b-PspA-C-CPE.

For protein expression, these plasmids were transformed into *Escherichia coli* strain BL21 (DE3) (TaKaRa BIO, Shiga, Japan). Isopropyl-β-d-thiogalactopyranoside (final concentration, 1 mM; Nacalai Tesque) was added to induce recombinant protein expression. The cells were harvested, suspended in phosphate-buffered saline (PBS; Nacalai Tesque), and disrupted by ultrasonication (45 seconds, 3 times). After insoluble material was removed through centrifugation (17,800 × *g*, 15 minutes, 4°C), the supernatant was filtered through a 0.45-μm Millex-HV filter unit (Merck Millipore, Burlington, MA, USA).

To purify the histidine-tagged fusion protein, the filtrate underwent affinity purification (NGC chromatography system [Bio-Rad, Hercules, CA, USA], HisTrap HP column [Cytiva, Tokyo, Japan]). PspA was dissolved in a buffer comprising 10 mM Tris-HCl [pH 8.0] (Nippon Gene, Tokyo, Japan), 400 mM NaCl (Nacalai Tesque), 5 mM MgCl_2_ (Nacalai Tesque), 0.1 mM phenylmethylsulfonyl fluoride (Nacalai Tesque), 1 mM 2-mercaptoethanol (Nacalai Tesque), and 10% glycerol (Nacalai Tesque). The eluted fractions were concentrated using a 30-kDa cut-off centrifugal filter unit (Merck Millipore), and the solvent was replaced with PBS.

Phase separation chromatography was used to remove endotoxin from the purified protein solution. Triton X-114 (final concentration, 1% [v/v]; Nacalai Tesque) was added to the purified protein solution, which then was vortexed for 30 seconds and incubated on ice for 5 minutes. Next, to induce phase separation, the solution was heated in a water bath at 37°C for 5 minutes. After centrifugation (3,000 × *g*, 3 minutes, 25°C), the upper layer containing the protein was collected. This procedure was performed three times in total.

After purification, the protein concentration was measured using the BCA Protein Assay Kit (Thermo Fisher Scientific, Waltham, MA, USA), and the endotoxin concentration was measured using the ToxinSensor Chromogenic LAL Endotoxin Assay Kit (GenScript Biotech, Piscataway, NJ, USA). Protein purity was confirmed by Coomassie Brilliant Blue staining after SDS-PAGE using the NuPAGE electrophoresis system (Invitrogen, Carlsbad, CA, USA).

### Immunization

For immunization via the sublingual route, isoflurane-anesthetized mice received either: (1) 5 μL PBS; (2) 5 μL PBS containing 5 μg PspA-C-CPE; or (3) 5 μL PBS containing 5 μg PspA-C-CPE and 1 μg *Alcaligenes* lipid A. To promote antigen absorption, mice were kept in a head-down position for 30 minutes after administration. This immunization protocol was performed three times at weekly intervals.

One week after the final immunization, samples were harvested and prepared. For serum samples, blood was collected and stored on ice and then centrifuged (3,000 × *g*, 10 minutes, 4°C) to separate the serum. Feces were weighed, PBS was added to generate a 100-mg/mL suspension, and the mixture was vortexed for 10 minutes; this mixture was centrifuged (3,000 × *g*, 10 minutes, 4°C) and the supernatant collected. Nasal wash fluid was obtained by incising the trachea of mice after they had been killed, perfusing 200 μL PBS through the nasal cavity and collecting the aspirate. Bronchoalveolar lavage fluid (BALF) was collected by injecting 1 mL PBS through the trachea into the lungs of mice after they had been killed and then flushing and absorbing the fluid five times via a cut in the trachea. All samples were stored at − 30°C until use.

### Detection of antigen-specific antibodies by ELISA

ELISA was used to measure antigen-specific antibody titers. PspA or C-CPE (each at 5 μg/mL in PBS) was added at 100 μL/well to a 96-well plate and incubated overnight at 4°C to coat the wells. The coating solution was removed, and the wells were blocked by adding PBS containing 1% BSA (Nacalai Tesque) and incubating them at room temperature for 2 hours. The wells then were washed three times with PBS containing 0.05% Tween 20 (Nacalai Tesque), after which serum, nasal wash, BALF, or fecal supernatant samples were added to the wells and incubated at room temperature for 2 hours. The wells again were washed three times, after which horseradish peroxidase-labeled goat anti-mouse IgG or IgA antibody (diluted 1:4,000; SouthernBiotech, Birmingham, AL, USA) was added and incubated at room temperature for 1 hour. The wells again were washed three times, tetramethylbenzidine substrate (SeraCare Life Sciences, Milford, MA, USA) was added, and the plates were incubated at room temperature for 2 minutes. The reaction was stopped with 0.5 M HCl, and absorbance was measured at 450 nm (iMark Microplate Reader; Bio-Rad).

### CPE cytotoxicity assay

Vero cells (5.0 × 10^4^ cells per well) were inoculated into a 96-well plate and cultured (5% CO_2_, 37°C). Two days after inoculation, CPE (0.1 μg) and sera from immunized mice (40 μL) were incubated for 1 h at 37°C, after which this CPE–serum mixture was added to each well and the plates incubated (5% CO_2_, 37°C) for 30 minutes. The wells were gently washed with PBS, and Cell Count Reagent SF (Nacalai Tesque) was added to each well to detect live cells. After 2 hours, absorbance was measured at 450 nm.

### CPE-induced hyperkalemia model and measurement of serum potassium

To evaluate the CPE infection-preventing effect of the vaccination strategy, mice were injected intravenously with CPE (100 μg/kg body weight; AUTiv^™^ Mouse Tail Vein Automatic Injection System; Natsume Seisakusho, Tokyo, Japan). Mice were videotaped 30 minutes after administration. At 3 hours after administration, isoflurane-anesthetized mice underwent cardiocentesis, and serum was separated from the harvested blood. Serum potassium concentrations were measured (Dri-Chem 7000 System; Fujifilm, Tokyo, Japan).

### S. pneumoniae culture and infection model

*S. pneumoniae* Xen10 was cultured overnight under non-aerobic conditions (at 37°C, 5% CO_2_) in brain–heart infusion broth (Becton, Dickinson and Company, Franklin Lakes, NJ, USA). *S. pneumoniae* was recovered by centrifugation (3,000 × *g*, 15 minutes, 4°C) and then washed twice by rinsing with PBS followed by centrifugation (9,100 × *g*, 3 minutes, 4°C). One week after the final immunization, mice were anesthetized and infected with the *S. pneumoniae* suspension (5×10^6^ CFU/40 μL) via intranasal administration. The survival rate and body weight changes of mice were monitored for 14 days after infection. Mice that lost 20% or more of their preinfection body weight were euthanized considering a humane endpoint.

#### S. pneumoniae CFU in lung

Infected mice were euthanized and their lungs removed and homogenized for 1 minute in 10 mL sterile PBS (Nacalai Tesque). Samples of homogenate were plated on blood agar plates (BD Biosciences) coated with kanamycin (40 μL, 100 μg/mL; Nacalai Tesque). The plates were incubated overnight at 37°C and the bacterial colonies counted.

### Statistical analysis

Data are presented as the mean ± SD. Statistical analyses were performed using one-way ANOVA with Tukey’s multiple comparison test after ROUT outlier identification (PRISM 10.1.2; GraphPad Software, San Diego, CA, USA). The threshold for statistical significance was defined as *P* < 0.05.

## Results

### Sublingual administration of bivalent antigen PspA-C-CPE with mucosal adjuvant Alcaligenes lipid A induced antigen-specific antibody production at mucosal surfaces

**Figure 1:**
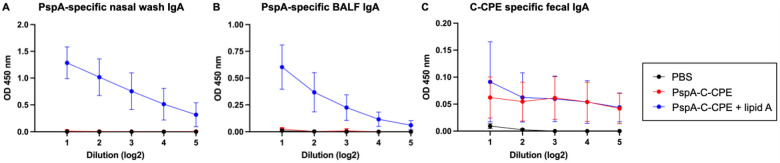
Sublingual administration of PspA-C-CPE with *Alcaligenes* lipid A enhances antigen-specific antibody production at mucosal surfaces. Mice were sublingually immunized with PBS, PspA-C-CPE only, or PspA-C-CPE combined with *Alcaligenes* lipid A three times at 1-week intervals. At 1 week after the final immunization, nasal wash, bronchoalveolar lavage fluid (BALF), and fecal extracts were collected (n = 8 per group). PspA-specific IgA in nasal wash (A), PspA-specific IgA in BALF (B), and C-CPE-specific IgA in fecal extracts (C) were measured by ELISA. Data are representative of two independent experiments and are presented as the mean ± SD.

**Figure 2:**
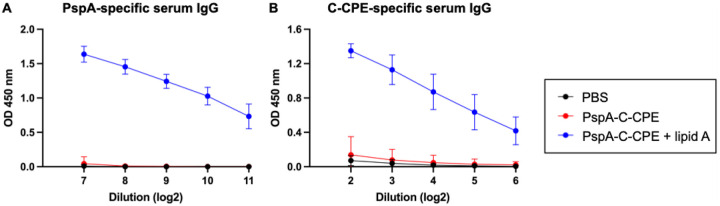
Sublingual administration of PspA-C-CPE with *Alcaligenes* lipid A enhances systemic antigen-specific antibody production. Mice were sublingually immunized with PBS, PspA-C-CPE only, or PspA-C-CPE combined with *Alcaligenes*lipid A three times at 1-week intervals. Serum samples (n = 8 per group) were collected 1 week after the final immunization. PspA-specific IgG (A), and C-CPE-specific IgG (B) levels in the serum were determined by ELISA. Data are representative of two independent experiments and are presented as the mean ± SD.

To investigate whether sublingual administration of the bivalent vaccine construct PspA-C-CPE with mucosal adjuvant *Alcaligenes* lipid A induces concurrent antigen-specific immune responses in the respiratory and gastrointestinal tracts, we evaluated antibody production in mice. Mice were sublingually immunized with PspA-C-CPE in the presence or absence of *Alcaligenes* lipid A, and mucosal antibody responses were assessed by ELISA. The levels of PspA-specific IgA and IgG in nasal washes and BALF and C-CPE-specific IgA in fecal extracts were comparable to or significantly higher than those in mice immunized with PspA-C-CPE alone (Fig. 1, Supplementary Fig. 1). We next examined the induction of systemic immune responses. Serum levels of PspA-specific IgG and IgA and C-CPE-specific IgG were significantly enhanced by the co-administration of *Alcaligenes* lipid A (Fig. 2, Supplementary Fig. 1). Collectively, these results indicate that sublingual immunization with PspA-C-CPE, using *Alcaligenes* lipid A as an adjuvant, is associated with enhanced PspA-specific antibody production in the respiratory tract and amplified systemic antibody responses against both antigens.

### Sublingual administration of PspA-C-CPE with Alcaligenes lipid A induced neutralizing antibodies against CPE and mitigated the severity of CPE intoxication

**Figure 3:**
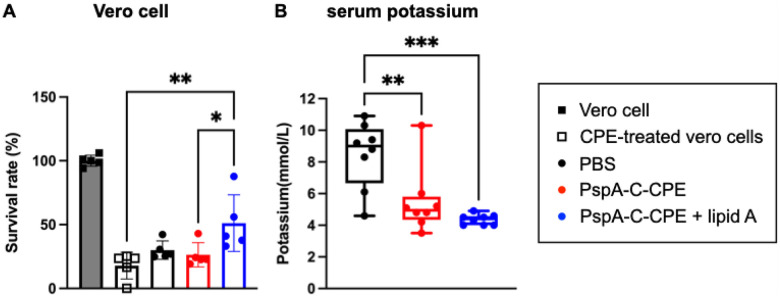
Sublingual administration of PspA-C-CPE with *Alcaligenes* lipid A inhibits CPE-induced hyperkalemia by inducing C-CPE-specific neutralizing antibodies. Mice were sublingually immunized with PBS, PspA-C-CPE only, or PspA-C-CPE combined with *Alcaligenes*lipid A three times at 1-week intervals. (A) Serum samples (n = 5 per group) were collected 1 week after the final immunization. The sera were mixed with CPE and added to Vero cells. Cell viability (%) was calculated based on the number of viable cells after 30 minutes of incubation. Data are presented as the mean ± SD. (B) One week after the final immunization, mice were intravenously injected with CPE (100 μg/kg body weight) via the tail vein (n = 8 per group). Serum samples were collected 3 hours after CPE challenge to measure potassium levels. The physiological range for serum potassium is 3.8–5.0 mmol/L. The boxes show the interquartile range, with the median value indicated by the horizontal line; whiskers show the range. Data are representative of two independent experiments. Statistical analysis was performed using one-way ANOVA followed by Tukey’s test (*P < 0.05, **P < 0.01, ***P < 0.001).

We next examined whether C-CPE-specific antibodies induced by sublingual immunization with PspA-C-CPE and *Alcaligenes* lipid A exhibit neutralizing activity against CPE. To evaluate the neutralizing capacity of serum IgG, we used Vero cells, which are highly sensitive to CPE due to the endogenous expression of claudin receptors. Exposure to CPE significantly reduced the viability of Vero cells (Fig. 3A). Notably, treatment with serum from mice immunized with PBS or PspA-C-CPE alone failed to inhibit this CPE-induced cytotoxicity. In contrast, serum from mice immunized with PspA-C-CPE combined with *Alcaligenes* lipid A effectively suppressed the cytotoxicity. These results suggest that sublingual immunization with PspA-C-CPE and *Alcaligenes* lipid A induces serum C-CPE-specific IgG with potent neutralizing activity against CPE.

In severe cases of *C. perfringens* food poisoning, CPE translocates from the damaged intestinal epithelium into the bloodstream and reaches the liver. There, it binds to claudin receptors, triggering massive potassium efflux from hepatocytes, which results in lethal hyperkalemia [2]. Given the potent neutralizing activity observed *in vitro*, we next evaluated whether vaccine-induced antibodies protected against CPE-induced hyperkalemia *in vivo*. After intravenous challenge with CPE, mice in the PBS-only group exhibited severe clinical signs, including limb paralysis and generalized muscle weakness, accompanied by markedly elevated serum potassium levels (> 5.0 mmol/L) indicative of hyperkalemia (Fig. 3B, Supplementary Fig. 2). Although immunization with PspA-C-CPE alone partially mitigated the severity of the hyperkalemia and reduced serum potassium levels compared with those in the PBS-only group, some mice still exhibited clinical symptoms, such as paralysis. In marked contrast, co-administration of *Alcaligenes* lipid A completely prevented these clinical signs. Furthermore, serum potassium levels in the PspA-C-CPE + *Alcaligenes* lipid A group remained within the physiological normal range (3.8–5.0 mmol/L) and were significantly lower than in the PBS-only group (Fig. 3B, Supplementary Fig. 2). These results indicate that sublingual immunization with PspA-C-CPE + *Alcaligenes* lipid A confers robust protection against *C. perfringens*-associated pathology by neutralizing CPE and preventing the onset of hyperkalemia.

### Sublingual administration of PspA-C-CPE with Alcaligenes lipid A protected mice against lethal S. pneumoniae respiratory infection

**Figure 4:**
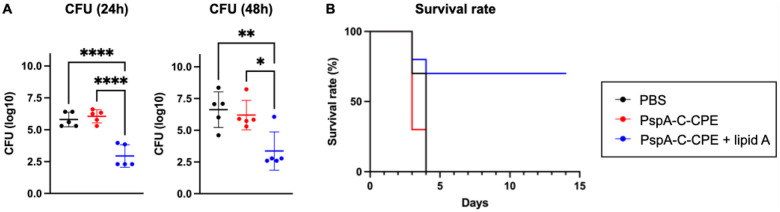
Sublingual administration of PspA-C-CPE with *Alcaligenes* lipid A protects against lethal *S. pneumoniae* respiratory infection. Mice were sublingually immunized with PBS, PspA-C-CPE only, or PspA-C-CPE combined with *Alcaligenes*lipid A three times at 1-week intervals. At 1 week after the final immunization, mice were challenged with a lethal dose of *S. pneumoniae*via the respiratory tract. (A) Bacterial burden (CFU) in the lungs. Lungs (n = 5 per group) were collected at 24 and 48 hours after infection. Lung homogenates were plated onto blood agar plates, and CFU counts were calculated as the number of colonies that formed after overnight incubation. Data are presented as the mean ± SD. (B) Survival rates were monitored daily for 14 days after infection (n = 10 per group). Data are representative of two independent experiments. Statistical analysis was performed using one-way ANOVA followed by Tukey’s test (*P< 0.05, **P < 0.01, ****P < 0.0001).

We next investigated whether sublingual immunization with PspA-C-CPE and *Alcaligenes* lipid A protected against respiratory infection by *S. pneumoniae*. At 1 week after the final immunization, mice were challenged with *S. pneumoniae* via the respiratory tract, and the bacterial burden in the lungs was evaluated by counting CFUs. At both 24 and 48 hours after infection, the bacterial load in the lungs of mice immunized with PspA-C-CPE and *Alcaligenes* lipid A was markedly reduced, approximately 1,000-fold lower than that observed in the PBS-only or PspA-C-CPE-only groups (Fig. 4A). This significant reduction suggests that the induction of functionally protective antibody responses facilitated the rapid clearance of *S. pneumoniae* from the lungs.

Building on these findings, we then assessed the survival of sublingually immunized mice exposed to a lethal respiratory challenge with *S. pneumoniae*. Mice in the PBS-only and PspA-C-CPE-only groups rapidly lost weight and succumbed to infection within 4–5 days (Fig. 4B, Supplementary Fig. 3). In contrast, survival rates were significantly improved in the group of mice sublingually immunized with PspA-C-CPE and *Alcaligenes* lipid A. Although these mice experienced transient body weight loss on days 2 and 3 after infection, they subsequently recovered this weight, and the majority survived the lethal challenge (Fig. 4B, Supplementary Fig. 3). These results suggest that sublingual immunization with PspA-C-CPE using *Alcaligenes* lipid A as an adjuvant confers robust protection against lethal *S. pneumoniae* respiratory infection, mediated by the induction of PspA-specific IgA and IgG responses.

### Sublingual administration of PspA-C-CPE with Alcaligenes lipid A induced sustained antigen-specific antibody responses

**Figure 5:**
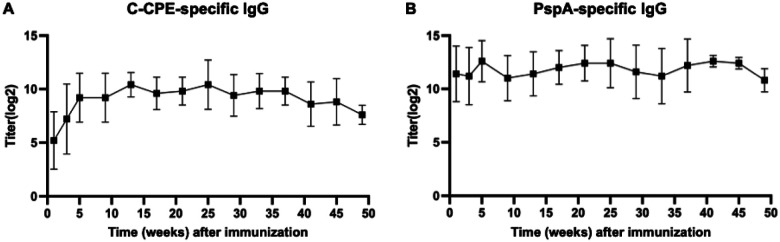
Sublingual administration of PspA-C-CPE with *Alcaligenes* lipid A induces long-lasting antigen-specific antibody production. Mice were sublingually immunized with PBS, PspA-C-CPE alone, or PspA-C-CPE combined with *Alcaligenes* lipid A three times at 1-week intervals. Serum samples were collected periodically for 49 weeks, starting 1 week after the final immunization. C-CPE-specific IgG (A) and PspA-specific IgG (B) levels were measured by ELISA (n = 5 per group). Titers were defined as the highest dilution factor yielding an optical density at 450 nm (OD_450_) greater than 0.5. Data are presented as the mean ± SD.

Finally, to assess the persistence of the immune responses enhanced by *Alcaligenes* lipid A, we monitored serum antibody levels against both C-CPE and PspA for an extended period. In mice sublingually immunized with PspA-C-CPE and *Alcaligenes* lipid A, high levels of serum IgG specific for both antigens were maintained for at least 49 weeks after the final immunization (Fig. 5). Specifically, C-CPE-specific IgG titers rose rapidly, peaked by week 5, and were sustained thereafter. Collectively, these results demonstrate that sublingual immunization with PspA-C-CPE, using *Alcaligenes* lipid A as an adjuvant, induces sustained antigen-specific antibody responses.

## Discussion

In the present study, we evaluated the potential of sublingual immunization using the recombinant bivalent fusion protein PspA-C-CPE, combined with *Alcaligenes* lipid A as a mucosal adjuvant. Specifically, this vaccination induced C-CPE-specific neutralizing antibodies, thereby effectively preventing the fatal sequela of CPE-induced hyperkalemia. Moreover, the vaccine elicited PspA-specific IgA and IgG with neutralizing activity, which accelerated the pulmonary clearance of *S. pneumoniae* and protected against respiratory infection. Together, these results indicate that our sublingual vaccination strategy effectively bridges mucosal and systemic immunity to neutralize toxins and clear bacterial colonizers. Consequently, we conclude that the PspA-C-CPE bivalent vaccine with concurrent mucosal (*Alcaligenes* lipid A) adjuvant administration offers a practical and versatile approach for providing comprehensive defense against multiple infectious diseases that present significant clinical challenges.

Sublingual administration of PspA-C-CPE combined with *Alcaligenes* lipid A successfully induced PspA-specific IgA production in the respiratory mucosa, a distant effector site. We previously reported that sublingual immunization with *Alcaligenes* lipid A promotes the proliferation of antigen-specific T cells and enhances IL-17 production in the draining lymph nodes [22]. In the context of mucosal immunity, Th17 cells play a pivotal role in both host defense against extracellular bacterial pathogens and in the induction of IgA responses. Specifically, IL-17 produced by Th17 cells upregulates the expression of the polymeric immunoglobulin receptor on epithelial cells, thereby facilitating the transcytosis of IgA to mucosal surfaces [25]. Furthermore, IL-17 has been shown to induce class-switch recombination to IgA in B cells [26]. We previously confirmed that, concurrently with these T cell responses, sublingual administration of *Alcaligenes* lipid A increases the frequency of germinal center (GC) B cells (CD45^+^B220^+^GL7^high^) in the draining lymph nodes [22]. The GC reaction is a critical process for antibody affinity maturation and the establishment of immunological memory, reflecting T cell-dependent B cell activation. The generation of high-affinity IgA requires the differentiation of B cells into plasma cells following somatic hypermutation and affinity maturation within GCs [27]. Collectively, these findings suggest that *Alcaligenes* lipid A promotes PspA-specific IgA production in the distant respiratory tract by inducing both Th17 cell responses and GC B cell formation in the draining lymph nodes, followed by the subsequent mucosal homing of these activated cells to the effector sites.

Regarding C-CPE, co-administration with *Alcaligenes* lipid A successfully induced the production of serum IgG antibodies. C-CPE is inherently a poorly immunogenic molecule deficient in T-cell epitopes; previous reports indicate that even when administered with adjuvants, it primarily elicits IgM responses, and inducing class-switched IgG antibodies is difficult [11]. We propose that the robust induction of C-CPE-specific IgG observed in the present study is due to the recruitment of T-cell help from the highly immunogenic PspA partner, a mechanism analogous to a bystander effect. As demonstrated in previous studies with the Shiga toxin 2 B subunit (Stx2B)-C-CPE [11], antigen-presenting cells that take up the fusion protein present T-cell epitopes derived from the partner antigen (PspA in the present study), thereby activating PspA-specific T cells. It is plausible that these activated T cells provide cognate help, via CD40 ligand and cytokines, to C-CPE-specific B cells that have internalized the same fusion protein. This interaction facilitates somatic hypermutation and class-switch recombination within the C-CPE-specific B cells, ultimately resulting in the efficient production of C-CPE-specific IgG [11].

The exacerbation of *C. perfringens* foodborne illness is primarily driven by the systemic pathogenesis resulting from the translocation of CPE into the bloodstream [2]. Specifically, CPE binds via its C-terminal domain to claudin-4 on the intestinal epithelium, followed by oligomerization of the N-terminal domain. This process leads to epithelial membrane disruption and pore formation, facilitating the toxin’s entry into the systemic circulation. Subsequently, the circulating toxin targets claudin-3 and claudin-4 expressed in the liver and kidneys, inducing cell death and ultimately causing lethal hyperkalemia [10]. Therefore, inhibiting the interaction between CPE and its receptors represents a rational preventive strategy to preclude the progression to severe disease. In the present study, in addition to suppressing CPE-induced cytotoxicity *in vitro*, C-CPE-specific serum IgG induced by sublingual immunization with PspA-C-CPE and *Alcaligenes* lipid A reduced the increase in serum potassium concentrations after intravenous CPE challenge *in vivo*. These findings suggest that C-CPE-specific IgG, elicited by the *Alcaligenes* lipid A-adjuvanted vaccine, effectively captures and neutralizes circulating CPE in the bloodstream. By blocking the binding of CPE to claudin receptors on target organs, these antibodies prevent the onset of lethal pathogenesis.

In addition to its efficacy against CPE, sublingual immunization with PspA-C-CPE and *Alcaligenes* lipid A demonstrated potent protective effects against *S. pneumoniae* infection. Given that *S. pneumoniae* comprises over 100 serotypes and that current vaccines offer limited serotype coverage, there is an urgent need for broad-spectrum vaccines targeting PspA, a surface protein that is highly conserved across clinical isolates and capable of eliciting cross-reactive immunity [5–8]. In the present study, mice sublingually immunized with PspA-C-CPE and *Alcaligenes* lipid A exhibited significant reductions in pulmonary bacterial burden and improved survival rates after lethal respiratory challenge. We attribute this robust protection to the adjuvant’s ability to enhance multiple arms of the immune system, including PspA-specific antibody production and Th17 responses. Specifically, PspA-specific IgA induced in the respiratory tract likely binds to *S. pneumoniae*, thereby inhibiting bacterial adhesion to the epithelium and preventing colonization [28]. Furthermore, PspA-specific IgG transudating from the systemic circulation into the alveolar space is presumed to neutralize the complement-inhibitory function of PspA, thereby promoting opsonophagocytosis. Moreover, considering that *Alcaligenes* lipid A acts as a Th17-inducing adjuvant, IL-17-mediated recruitment of neutrophils to the site of infection likely contributed to the rapid clearance of *S. pneumoniae* [29].

In the present study, assessment of the durability of antigen-specific antibody production revealed that IgG titers remained elevated for approximately 49 weeks after the final immunization. Generally, the long-term maintenance of serum antibody levels is sustained by long-lived plasma cells (LLPCs) that reside in survival niches, such as the bone marrow [30]. Differentiation into LLPCs requires affinity maturation and class-switch recombination within GCs [31]. We previously demonstrated that the addition of *Alcaligenes* lipid A as a sublingual adjuvant significantly increased the frequency of GC B cells in the draining lymph nodes [22]. The sustained antibody production observed over 49 weeks in the present study suggests that robust GC reactions induced by *Alcaligenes* lipid A facilitated the selection of high-affinity B cells and their efficient differentiation into LLPCs. Notably, C-CPE-specific IgG titers continued to rise even after the completion of the immunization schedule. We hypothesize that this phenomenon is driven by the sustained provision of help from PspA-specific T cells. This ongoing T-cell help likely promotes class switching and differentiation of C-CPE-specific B cells into LLPCs, thereby enhancing and maintaining IgG production against C-CPE [11].

Although we have demonstrated the efficacy of our sublingual vaccination strategy using PspA-C-CPE and *Alcaligenes* lipid A in this study, several limitations remain to be addressed. First, the precise mechanism of C-CPE-mediated antigen delivery within the sublingual mucosa requires further elucidation. Although C-CPE fusion proteins are postulated to facilitate efficient antigen delivery and subsequent immune activation by targeting claudin-4 [11, 12, 24], we did not directly demonstrate the *in vivo* binding of PspA-C-CPE to claudin-4 in the sublingual epithelium in the present study. Therefore, future histological investigations are warranted to track the spatiotemporal localization and uptake dynamics of the antigen following sublingual administration. Second, regarding the mechanisms underlying PspA-specific T-cell help for C-CPE-specific IgG induction and the capacity of *Alcaligenes* lipid A to induce Th17 cells, we have not yet performed detailed functional analyses of antigen-specific T cells or comprehensive cytokine profiling. These aspects require further investigation to fully elucidate the immunological pathways involved. Third, although this vaccine strategy successfully induced systemic C-CPE-specific IgG with potent neutralizing activity, C-CPE-specific IgA levels in the intestinal mucosa were low. Given that CPE initially acts at the intestinal epithelial surface, further improvement of mucosal IgA induction against C-CPE is desirable to strengthen local protection at the site of toxin action. One possible strategy is to enhance the antigenicity of C-CPE by multimerizing C-CPE domains or incorporating repeated C-CPE units within the fusion antigen. Such structural optimization may increase B-cell receptor engagement and improve the magnitude of C-CPE-specific mucosal antibody responses. In addition, optimizing the antigen configuration, linker design, or adjuvant dose may further enhance intestinal IgA induction. Future studies should therefore focus on improving the antigen design to elicit more robust C-CPE-specific IgA responses while maintaining the systemic neutralizing IgG response observed in the present study. Fourth, although the long-term maintenance of antigen-specific IgG strongly suggests the induction of LLPCs, we have not directly verified their establishment in the bone marrow, which serves as their primary survival niche. Finally, the breadth of cross-protection warrants further validation. Our current infection model was limited to a single *S. pneumoniae* strain; therefore, a comprehensive evaluation of efficacy against diverse pneumococcal serotypes is essential to confirm the vaccine's broad-spectrum potential. Moving forward, we aim to optimize *Alcaligenes* lipid A as a versatile vaccine adjuvant (e.g. by enhancing its functionality and reducing the effective dosage for practical application). Concurrently, we plan to conduct further immunological analyses and extended infection experiments to resolve these limitations, ultimately advancing the clinical translation of this promising mucosal vaccine platform.

In conclusion, we demonstrated that sublingual immunization of mice with the bivalent PspA-C-CPE vaccine with *Alcaligenes* lipid A as an adjuvant successfully confers simultaneous protection against both *S. pneumoniae* and *C. perfringens*. This single-antigen strategy provides a highly practical framework for multivalent vaccine development, substantially reducing manufacturing costs and simplifying quality control compared with conventional multi-antigen admixtures. From a public health perspective, effectively bridging mucosal and systemic immunity offers an advantage: it has the potential to suppress pneumococcal nasopharyngeal carriage and subsequent community transmission while simultaneously neutralizing CPE to preclude severe, outbreak-associated foodborne illness in communal settings. Finally, the marked persistence of the antibody response, coupled with the non-invasive nature of sublingual administration, holds immense clinical value. For vulnerable populations, such as the elderly in nursing homes or patients receiving home care, this regimen can provide a sustained immune response against perennially occurring pathogens without the need for frequent or invasive medical interventions. Ultimately, our study highlights the profound potential of *Alcaligenes* lipid A-adjuvanted PspA-C-CPE as a next-generation mucosal vaccine platform for comprehensive infectious disease control.

## Supplementary Files

This is a list of supplementary files associated with this preprint. Click to download.

- SupplementaryFigures.docx
- SupplementaryFigure2PspACCPElipidA.mp4
- SupplementaryFigure2PspACCPE.mp4
- SupplementaryFigure2PBS.mp4
- GraphicalAbstract.png

## Data Availability

The datasets used and/or analysed during the current study are available from the corresponding author on reasonable request.
